# Transcriptome profiling of two maize inbreds with distinct responses to Gibberella ear rot disease to identify candidate resistance genes

**DOI:** 10.1186/s12864-018-4513-4

**Published:** 2018-02-09

**Authors:** Aida Z. Kebede, Anne Johnston, Danielle Schneiderman, Whynn Bosnich, Linda J. Harris

**Affiliations:** 1 Ottawa Research and Development Centre (ORDC), Agriculture and Agri-Food Canada, 960 Carling Ave, Ottawa, ON K1A 0C6 Canada; 2 Morden Research and Development Centre, Agriculture and Agri-Food Canada, 101 Route 100, Morden, MB R6M 1Y5 Canada

**Keywords:** Maize, Gibberella ear rot, Candidate gene, RNA-Seq, QTL, Transcriptome, *Fusarium graminearum*, Gene expression, Disease resistance, Fungal pathogen

## Abstract

**Background:**

Gibberella ear rot (GER) is one of the most economically important fungal diseases of maize in the temperate zone due to moldy grain contaminated with health threatening mycotoxins. To develop resistant genotypes and control the disease, understanding the host-pathogen interaction is essential.

**Results:**

RNA-Seq-derived transcriptome profiles of fungal- and mock-inoculated developing kernel tissues of two maize inbred lines were used to identify differentially expressed transcripts and propose candidate genes mapping within GER resistance quantitative trait loci (QTL). A total of 1255 transcripts were significantly (*P* ≤ 0.05) up regulated due to fungal infection in both susceptible and resistant inbreds. A greater number of transcripts were up regulated in the former (1174) than the latter (497) and increased as the infection progressed from 1 to 2 days after inoculation. Focusing on differentially expressed genes located within QTL regions for GER resistance, we identified 81 genes involved in membrane transport, hormone regulation, cell wall modification, cell detoxification, and biosynthesis of pathogenesis related proteins and phytoalexins as candidate genes contributing to resistance. Applying droplet digital PCR, we validated the expression profiles of a subset of these candidate genes from QTL regions contributed by the resistant inbred on chromosomes 1, 2 and 9.

**Conclusion:**

By screening global gene expression profiles for differentially expressed genes mapping within resistance QTL regions, we have identified candidate genes for gibberella ear rot resistance on several maize chromosomes which could potentially lead to a better understanding of *Fusarium* resistance mechanisms.

**Electronic supplementary material:**

The online version of this article (10.1186/s12864-018-4513-4) contains supplementary material, which is available to authorized users.

## Background

Gibberella ear rot (GER) is a fungal disease of maize ears caused by *Fusarium graminearum*. Fungal airborne spores either infect by landing on exposed silks and growing down the silks to the developing kernels or directly through wounds to the ear caused by insects or extreme weather events [1]. The pathogen also causes stalk and root rot of maize and has a wide range of other cereal crop hosts such as wheat, barley, rice, and oats, thus affecting the productivity of the most important food crops cultivated in the temperate zone [2]. GER epidemics in maize may be sporadic as successful disease infection and establishment is contingent on specific environmental conditions within a short window of time, specifically high rainfall or humidity coupled with warm temperatures (24–28 °C) during the 10–15 days after silking [3]. Despite the erratic occurrence of epidemics, the fact that the pathogen contaminates grain with harmful mycotoxins [1, 4] emphasizes the need to control the disease.

Development of GER resistant genotypes is the most recommended method of disease control since the use of fungicides and cultural practices are less effective [1]. Genotypes with complete resistance have never been reported but genotypes that could retard the growth and development of the disease and consequently reduce disease severity and mycotoxin level at harvest have been described [4]. The mechanism by which these genotypes resist the disease is highly complex and yet to be fully understood. So far, studies have indicated that, despite considerable environmental effects on resistance, quantitative trait loci (QTL) conferring resistance have been detected on various chromosomes but stable QTL over different genetic backgrounds are limited [1, 5–7]. Moreover, breeding progress or selection gains had been slow due to the requirement of intensive field work and multi-environment testing.

To improve breeding efficiency for GER resistance and understand host-pathogen interaction at the molecular level, advances in the next generation sequencing technologies offer powerful tools. Applying genotyping-by-sequencing [8] to 410 B73 X CO441 F_6_ recombinant inbred lines, we recently mapped GER disease resistance QTL using 931 filtered haplotype tagging SNPs and significantly improved the accuracy and precision of identified chromosomal regions [9]. Notably, the QTL regions were flanked by a much narrower marker-to-marker distance which could in turn significantly reduce unwanted linkage drag when performing marker-assisted selections. These QTL could be even more useful if we could identify which of those genes within the QTL regions are involved in the resistance.

RNA-Seq analysis of differential gene expression could serve as an alternative method to discover candidate genes together with the QTL regions we recently identified. The state of a given cell/tissue can be captured through the detection of the transcribed RNAs at any time point. RNA-Seq provides whole genome scanning of expressed genes, including splice variants, uncalled genes, and noncoding RNAs [10].

*F. graminearum* is characterized as a hemibiotrophic fungal pathogen, initially colonizing live host tissue as a parasite and continuing to flourish even after host tissue cell death [11]. Programmed cell death or the hypersensitive response in plants is designed to deprive the pathogen of its resources and localize the infection by killing the cells surrounding it. However, the hypersensitive response has been reported to exacerbate the spread of infection by hemibiotrophic pathogens [12]. Secondary metabolites such as phenolics, phytoalexins and anti-microbial proteins have been suggested as mechanisms of resistance [13, 14], but the regulation and identification of genes involved in the biosynthesis of the secondary metabolites is still an active area of research. One example of recently identified phytoalexins is the zealexins, sesquiterpenoids biosynthesized by maize during pathogen attack through the activity of the terpene synthases ZmTPS6 and ZmTPS11 [14].

The objectives of the current study were therefore to identify differentially expressed genes (DEG) between fungal and mock (sterile fungal medium only) inoculated maize ears and compare the DEG between a susceptible and a resistant inbred line. The DEG were characterized based on their gene ontology to understand their functionality and propose effective disease resistance mechanisms. We further assessed the possibility of detecting candidate genes by exploring the DEG within the recently identified silk and kernel GER resistance QTL regions and validating their gene expression profiles by quantitative PCR.

## Methods

### Plant material and field experimental conditions

Plant materials used in the current study were two inbreds with contrasting GER resistance. B73 is a GER susceptible inbred which also served as the reference genome for maize [15] and for which seed stock has been maintained at the Ottawa Research and Development Centre (ORDC), Agriculture and Agri-Food Canada for decades. CO441 is an inbred developed at the ORDC with good silk and kernel GER resistance [16].

For the RNA-Seq experiment, the two inbred lines were inoculated under field conditions over the years 2004 and 2006, with overhead irrigation to promote disease development. All field experiments were conducted at the Ottawa RDC experimental field station located at 45°23′N, 75°43′W and at an altitude of 93 m above sea level. Annual mean maximum and minimum temperatures for the growing period, i.e. summer season, were 28 °C and 15 °C, respectively. Average annual rainfall was 940 mm [17].

### Disease inoculum preparation and inoculation

We used a single *Fusarium graminearum* strain DAOM180378 for inoculation, obtained from the Canadian Collection of Fungal Cultures, Agriculture and Agri-Food Canada, Ottawa, ON. We prepared the inoculum using a modified version of the method used by Reid et al. [13] and as described by Xue et al. [18]. Macroconidia were scraped from plates by pouring 10 ml Bilay’s (2 g KH_2_PO_4_, 2 g KNO_3_, 1 g dextrose, 1 g MgSO_4_, 1 g KCl, and 0.2 mg each of FeCl_3_, MnSO_4_, and ZnSO_4_ in 1 L water) per plate and subsequently straining through cheese cloth. The fungal inoculum was adjusted to 5 × 10^5^ spores/mL just prior to use.

Maize plants were selected based on their similarity in days to silking in each of the experimental years, to ensure uniformity in stage of kernel development and avoid environmental influence when we compare gene expression between fungal and mock treatments of the same inbred. For the RNA-Seq experiment, 6–8 sib-crossed primary ears from each inbred were inoculated with 1 ml of fungal inoculum on the same day prior to 10 am and a similar numbers of ears were injected with Bilay’s media as a mock treatment. Inoculation was performed 11 days after controlled pollination using the kernel inoculation method [19]. Three to four ears were then harvested 1 day and 2 days after inoculation. The harvested ears were placed in liquid nitrogen immediately to avoid RNA degradation, developing kernels ground in a mortar and pestle with liquid nitrogen and stored at − 80 °C until RNA extraction. In 2013, a similar procedure of inoculation and tissue sampling up to 5 days (including collecting untreated ears at the same developmental stages) was used to obtain samples for the validation experiment. RNA was extracted in bulk (3–4 ears per treatment, per testing year) from 2004 and 2006 field samples for the RNA-Seq and initial ddPCR experiments while individual ears from the 2013 field season were used as biological replicates for further ddPCR validation.

### RNA extraction and sequencing

High quality RNA was isolated using guanidine isothiocyanate and ultra-centrifugation with CsCl (cesium chloride) [20]. Total RNA quality was verified with a 2100 BioAnalyzer (Agilent Technologies) using the RNA 6000 Nano kit. Samples were then sent to the National Research Council Canada (Saskatoon, SK) for library construction (Illumina TruSeq RNA library prep kit v2) and RNA sequencing using Illumina HiSeq 2000 (Illumina Inc.). Paired end RNA sequence reads of 101 bases were generated for further analysis.

### RNA-Seq data analysis

The RNA-Seq data was analyzed using CLC Genomics workbench version 9 (Qiagen Corp.). Prior to the mapping and gene expression analysis, the raw data was trimmed based on quality scores that were determined by the base caller error probability level (*P* < 0.01). Sequences with very low score and with length less than 15 bases were discarded. To estimate the expression levels, the trimmed RNA sequences were aligned to the B73 reference genome version 2 annotated with genes and transcripts [15]. Read alignment was performed using the criteria: similarity and length fraction = 0.8, mismatch cost = 2, deletion and insertion costs = 3 and maximum number of hit per read = 10. Gene expression levels were estimated as transcripts per million (TPM) [21] which was calculated as: *TPM = (RPKM × 10*^*6*^*) / Σ RPKM*, where the sum is over the *RPKM* values of all genes/transcripts. Reads per kilobase of exon model per million mapped reads (RPKM) [10] was calculated following the formula: RPKM = total exon reads / [mapped reads (millions) × exon length (KB)].

An empirical analysis of differential gene expression or the ‘exact test’ according to Robinson and Smyth [22] was implemented to compare mock versus fungal inoculations. The ‘exact test’, a package also incorporated in EdgeR Bioconductor [23], is similar to Fisher’s exact test but also accounts for over dispersion caused by biological variability which makes it most suited for RNA-Seq data that has few biological replicates per experimental group.

Transcripts were considered as significantly differentially expressed when fold change was ≥2.0, False Discovery Rate (FDR) [24] corrected *P* ≤ 0.05 among groups and TPM ≥ 5 in at least one of the groups compared.

Functional enrichment analysis of significantly up regulated genes were performed using one of the g:Profiler web tool set, g:GOSt [25, 26]. In this method of gene ontology profiling, different ontology terms under biological processes, cellular components and molecular functions were enriched. The most significant ontology terms corresponding to these set of genes were identified using cumulative hypergeometric *P*-value estimates [25]. Additional interpretation of the significantly up and/or down regulated genes was performed through visualization of the genes’ involvement in known metabolic pathways and other biological processes using MAPMAN software [27] and the genome visualization tool Circos [28].

### Digital PCR validation experiment

We validated selected candidate gene expression profiles of fourteen genes using RNA from the same 2004 and 2006 samples as was used for RNA-Seq. To validate gene expression patterns over a broader number of time points and treatments, we sampled three biological replicates of B73 and CO441 developing kernels under untreated, mock- and fungal-inoculated conditions (1, 2, 4 and 5 DAI [days after inoculation]) in the 2013 field season.

Primers were designed for the selected gene transcripts (Additional file 1: Table S1) using the IDT PrimerQuest tool which incorporates Primer3 software (version 2.2.3) [29]. To check the specificity of the selected primers, forward and reverse primer sequences were submitted to the National Center for Biotechnology Information (NCBI) Blast program [30] to search nucleotide databases. Primers were designed to avoid transcript regions which contained insertions or deletions between inbreds, as visualized in RNA-Seq alignment data. In addition, primers were located within regions that were common between the different transcript isoforms and represented the overall gene expression.

Droplet digital PCR (ddPCR) was used to quantify and compare the expression levels of selected transcripts between untreated, mock and fungal treated kernel tissue samples. ddPCR uses emulsion chemistry to partition 20 μL samples into 20,000 oil encapsulated nanodroplets. This high level of partitioning serves to significantly increase precision and sensitivity while eliminating many of the requirements for optimization and validation that is associated with standard quantitative PCR. An average of five housekeeping genes, namely *FPGS* (folylpolyglutamate synthetase 1), *LUG* (transcriptional corepressor LEUNIG), *MEP* (membrane protein), *UBCE* (ubiquitin-conjugating enzyme) and *PGM* (phospoglucomutase), were used to normalize the ddPCR expression data (Additional file 1: Table S1). The housekeeping genes were selected based on their uniform expression in our datasets despite genotype or treatment difference, as suggested by Manoli et al. [31].

cDNA was synthesized using 800 ng of total RNA and iScript Reverse Transcription Supermix for RT-qPCR (Bio-Rad Laboratories Canada Ltd.) as described by the manufacturer. Each 25 μl ddPCR reaction contained 4 μl of a 10-fold cDNA dilution, 12.5 μl 2X QX200 ddPCR EvaGreen Supermix, 80 nM of each primer and DNase-free water. A no-template control was added for each primer pair. Each reaction (20 μl) along with droplet generator oil was transferred to the DG8 Cartridge onto the QX200 Droplet Generator (Bio-Rad Laboratories Canada Ltd) to generate the droplets as per manufacturer’s instructions. The droplets were transferred to a TwinTec 96-well PCR plate (Eppendorf Canada Ltd.) and cycled on a C1000 Touch Thermal Cycler (Bio-Rad Laboratories Canada Ltd.) using the default EvaGreen Supermix amplification protocol at an annealing temperature of 58 °C for each primer pair. Optimal annealing temperature was determined by running a temperature gradient for each primer pair ranging from 55 °C to 61 °C. Following amplification, the reaction plate was loaded on the QX200 Droplet Reader for detection. Data was analyzed using the instrument’s software (QuantaSoft version 1.6.6.0320, Bio-Rad Laboratories Canada Ltd.), and imported into Microsoft Excel for further analysis and normalization.

## Results

**Fig. 1:**
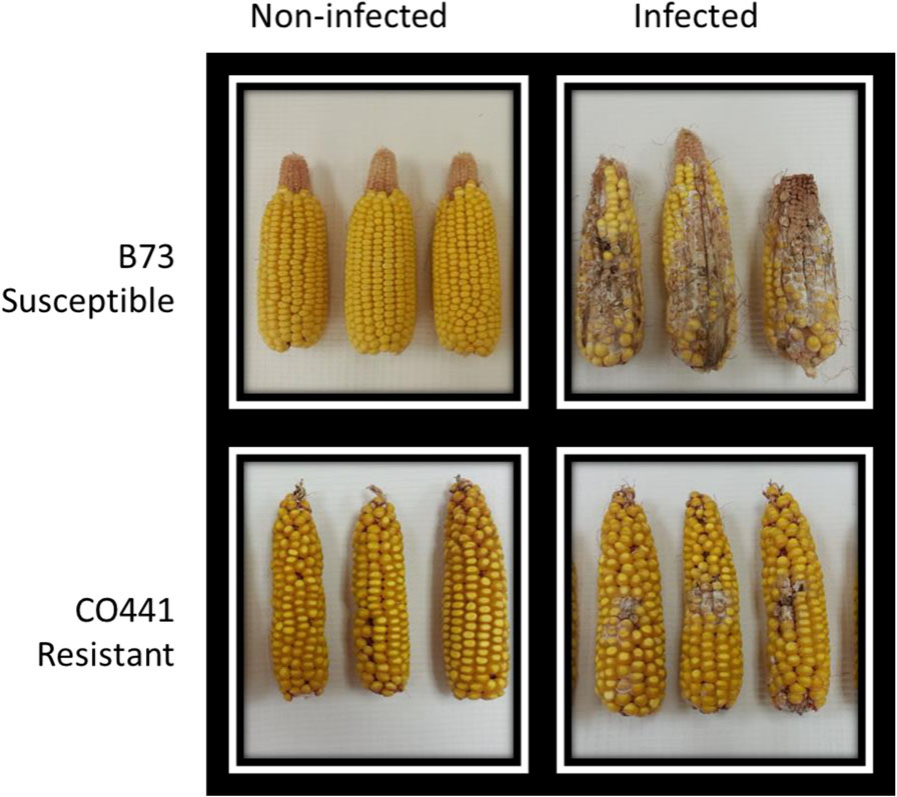
Disease severity levels observed in inbreds B73 and CO441. Sibcrossed ears 8 weeks after *F. graminearum* kernel inoculation alongside non-infected controls

Developing kernels of two inbred lines with contrasting gibberella ear rot resistance (Fig. 1) were fungal inoculated and sampled at two early time points during disease development (1 and 2 DAI). Although inoculation dates differed between the two inbreds by 16–18 days, climatic conditions were quite similar during the disease development periods and irrigation enhanced humidity levels (Additional file 1: Table S2). To identify differentially expressed genes (DEG), these samples were compared with mock inoculated samples using RNA-Seq. The experiment was conducted with at least three or four biological replicates per experimental group in each of the two testing years, 2004 and 2006. RNA extractions were performed from pooled tissues samples for each of the testing years and hence each year was considered as a biological replicate for data analysis.

### Read alignment and differential expression

On average, 27.6 million reads of up to 101 bases in length were obtained from each sample with slightly higher number of reads for B73 (28.6 million) than CO441 (26.7 million) (Additional file 1: Table S3). The reference genome B73 version 2 was used for read alignment and TPM was used for estimation of gene expression levels [21]. Proportions of reads aligned to the reference genome were similar for both B73 (95.68%) and CO441 (95.69%). This may be due to our less than stringent alignment parameters and some genomic divergence between our B73 seed stock and the source of the B73 genome sequence. From a total of 63,074 annotated transcripts [32], 72% were expressed in both inbred lines while 4% and 5% were expressed uniquely in B73 and CO441, respectively.

**Fig. 2:**
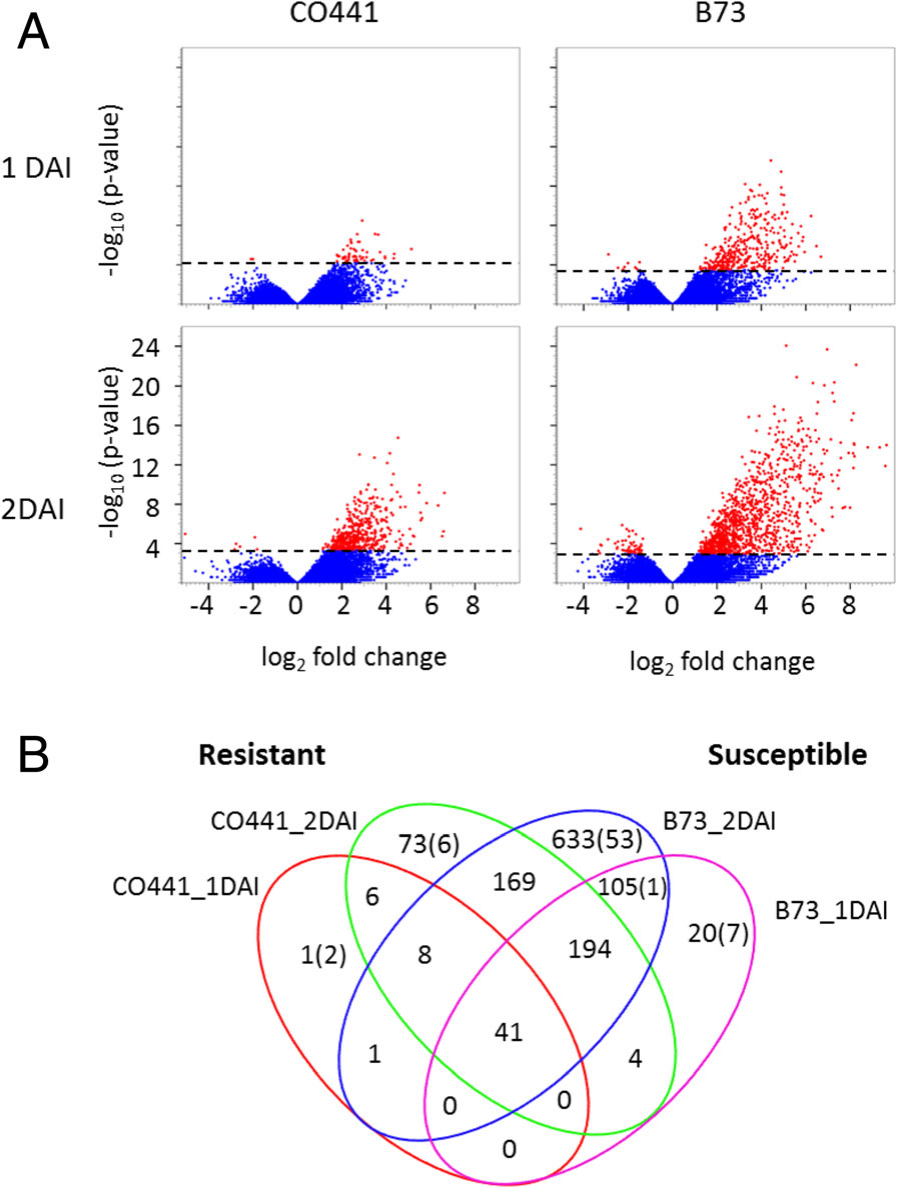
Differentially expressed transcripts in resistant and susceptible inbred lines. (**a**) The Log_2_ fold change highlighted in red shows significantly up or down regulated transcripts (FDR *P* < 0.05) after fungal inoculation relative to mock inoculation. (**b**) Venn diagram displaying differentially expressed transcripts unique and common between B73 and CO441. Down regulated transcripts are in brackets

Our analysis revealed 1324 transcripts (representing 1223 unique genes) with a significant (FDR, *P* ≤ 0.05 and ≥2 fold change) response to fungal infection of which 1255 were up regulated in either of the inbred lines tested (Fig. 2; Additional file 1: Table S4). The susceptible inbred B73 showed more up regulated transcripts (1174) than the resistant inbred CO441 (497). Around 33.1% of these transcripts were commonly up regulated while 60.4% were unique for B73 and 6.5% were unique for CO441. However, many of these genes were expressed at higher levels under mock inoculated conditions in CO441 relative to B73. For example, at 1 day after mock inoculation, there were 150 transcripts expressed greater than two-fold in CO441 versus B73 while only 25 transcripts were significantly increased in B73 relative to CO441 (FDR, *P* ≤ 0.05). As expected, fungal responsive genes increased in number as the infection progressed from 1 to 2 DAI.

### Functional enrichment

**Fig. 3:**
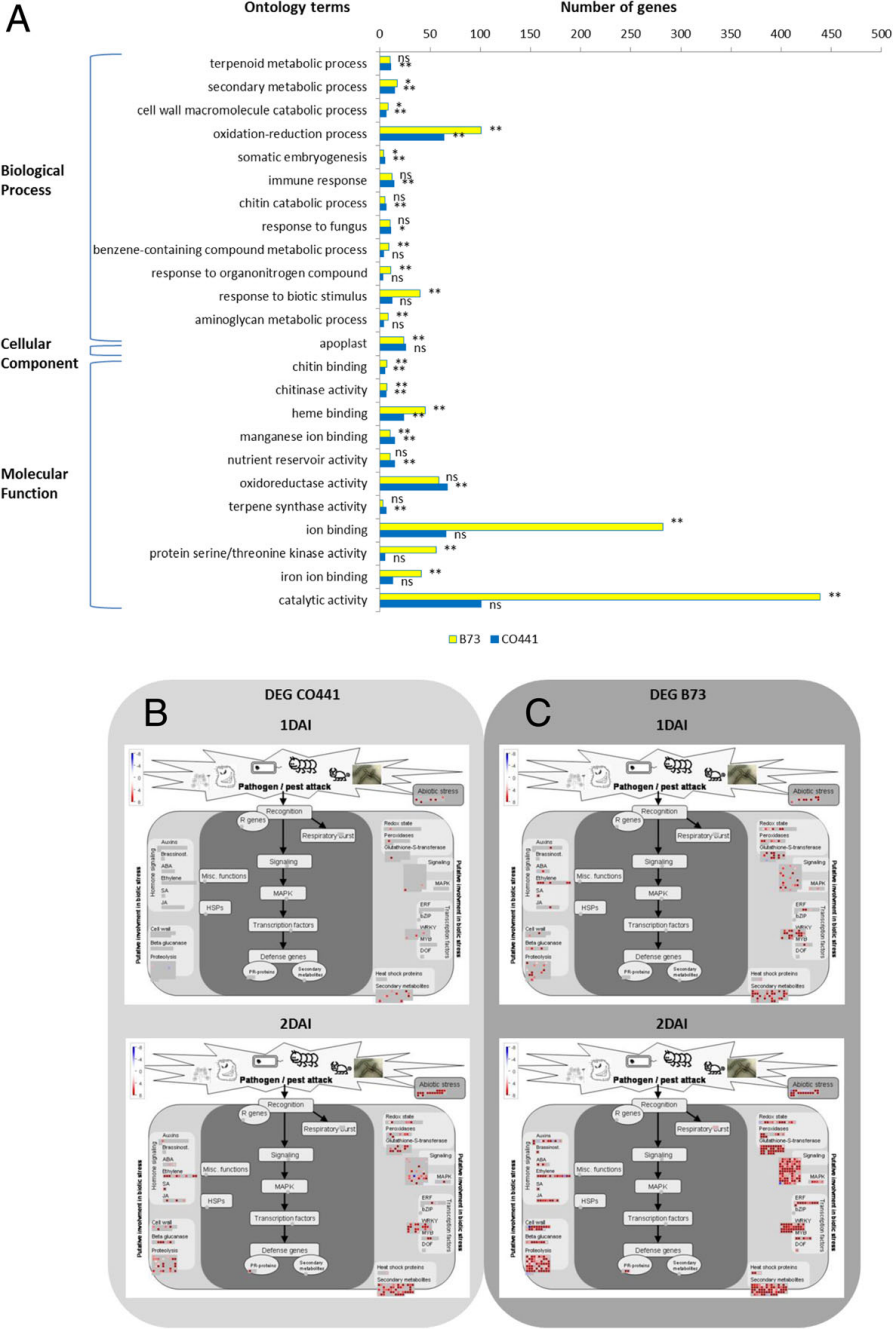
Annotation of genes induced after fungal inoculation. (**a**) Gene ontology terms associated with differentially expressed genes in B73 (yellow) and CO441 (blue). *, and ** significant at 0.05, and 0.01 probability levels, respectively. ns = non-significant. (**b** ,**c**) Mapman view of biotic stress pathway using significantly differentially expressed genes in CO441 (**b**) and B73 (**c**) at 1 and 2 DAI

We assigned the DEG to functional categories based on gene ontology and assessed their representation for each treatment relative to their representation in the genome. The apoplastic region, which includes the cell wall and extracellular space outside the cell membrane, is the initial site of interaction between pathogen and host (Fig. 3). The timing of subsequent molecular defence reactions differed between inbreds. This was observed through earlier induction in B73 than CO441 of gene transcripts involved in hormonal signaling which induced different transcription factors and biosynthesis of defensive anti-microbial proteins and other secondary metabolites collectively known as phytoalexins.

In the more resistant inbred CO441, genes involved in secondary metabolism, cell wall biosynthesis/modification and chitin catabolism, oxidation reduction processes, immune response and nutrient reservoir activity were significantly up-regulated (*P* < 0.01, Fig. 3a). From a Mapman view of biotic stress responses, we observed in CO441 an onset of secondary metabolism gene expression at 1 DAI which was followed by defence response signaling involving the hormones auxin, ABA, ethylene, salicylic and jasmonic acids at 2DAI (Fig. 3b and c). Heat shock proteins, MAPK signaling and PR-proteins and transcription factors – ERF, WRKY and MYB were also observed at 2DAI. For example, we identified 29 differentially expressed genes containing WRKY domains [33]; those significantly induced greater than five-fold after fungal challenge numbered 27 in B73 and 13 in CO441. Two WRKY genes (*GRMZM2G063880* and *GRMZM2G057116*) were expressed much higher in CO441 than B73 after mock inoculation (>5X; FDR, *P* < 0.05) and are also expressed at higher levels in a maize inbred resistant to *Aspergillus flavus* infection relative to the susceptible B73 [34]. The susceptible inbred B73 on the other hand, appeared to show similar biotic stress responsive genes at 1 DAI similar to what had been observed in CO441 at 2DAI. This was followed by more up regulated genes in each of the biotic stress response categories including PR proteins and respiratory burst or hypersensitive responses at 2 DAI suggesting earlier host cell death in the susceptible relative to the resistant inbred.

### Candidate gene identification

**Fig. 4:**
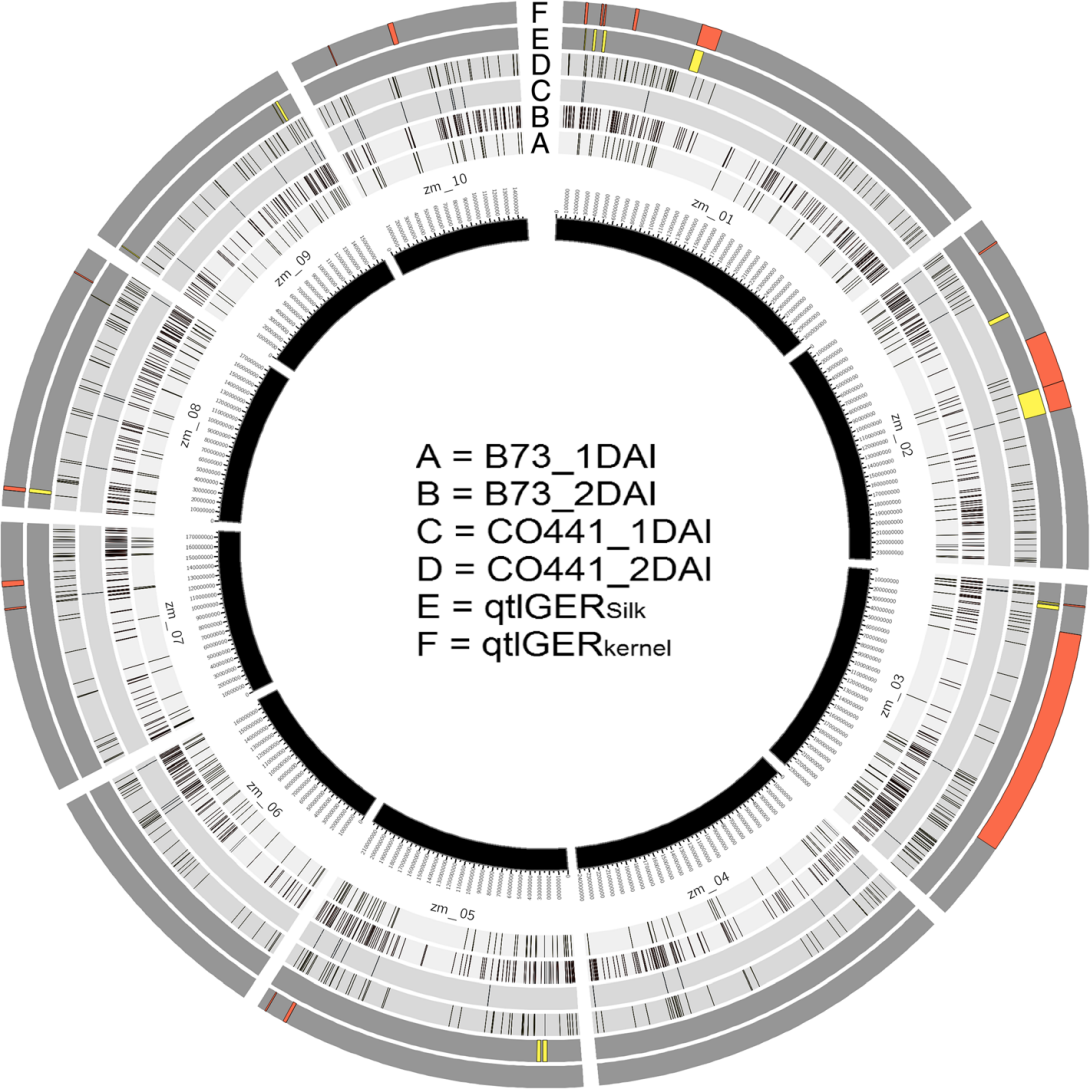
Distribution of DEG and GER resistance QTL regions across the maize genome. Circos was used to show significant differentially expressed genes (A-D) (fold change ≥2.0, FDR *P* < 0.05) and QTL regions detected for silk (E) and kernel (F) GER resistance

To identify genes with potential relevance to disease resistance, we screened for the presence of DEG within the recently mapped GER resistance QTL regions identified within a B73 X CO441 recombinant inbred population [9]. We found 87 up regulated and 10 down regulated gene transcripts within QTL regions for GER resistance towards either silk or kernel inoculation methods on chromosomes 1, 2, 3, 5, 7, 8 and 9 (Fig. 4; Additional file 1: Table S5). We focused on up regulated DEG representing 29 genes localized within QTL regions contributed by the resistant inbred CO441.

### Validation of candidate gene expression

To validate DEG expression profiles, we selected 14 genes that were significantly up regulated and detected within QTL regions on maize chromosomes 1, 2, and 9 (Additional file 1: Table S5). Using RNA from the same 2004 and 2006 samples, droplet digital PCR quantification of the selected DEGs and their RNA-Seq TPM values showed similar trends of expression in 12 of the 14 DEGs tested. However, two (*AC208897.3_FG004* and *GRMZM2G119150*) exhibited very low gene expression by ddPCR (despite testing multiple sets of primers) and appeared to show inconsistent expression patterns between the two methods of gene transcript quantification (Additional file 2).

**Fig. 5:**
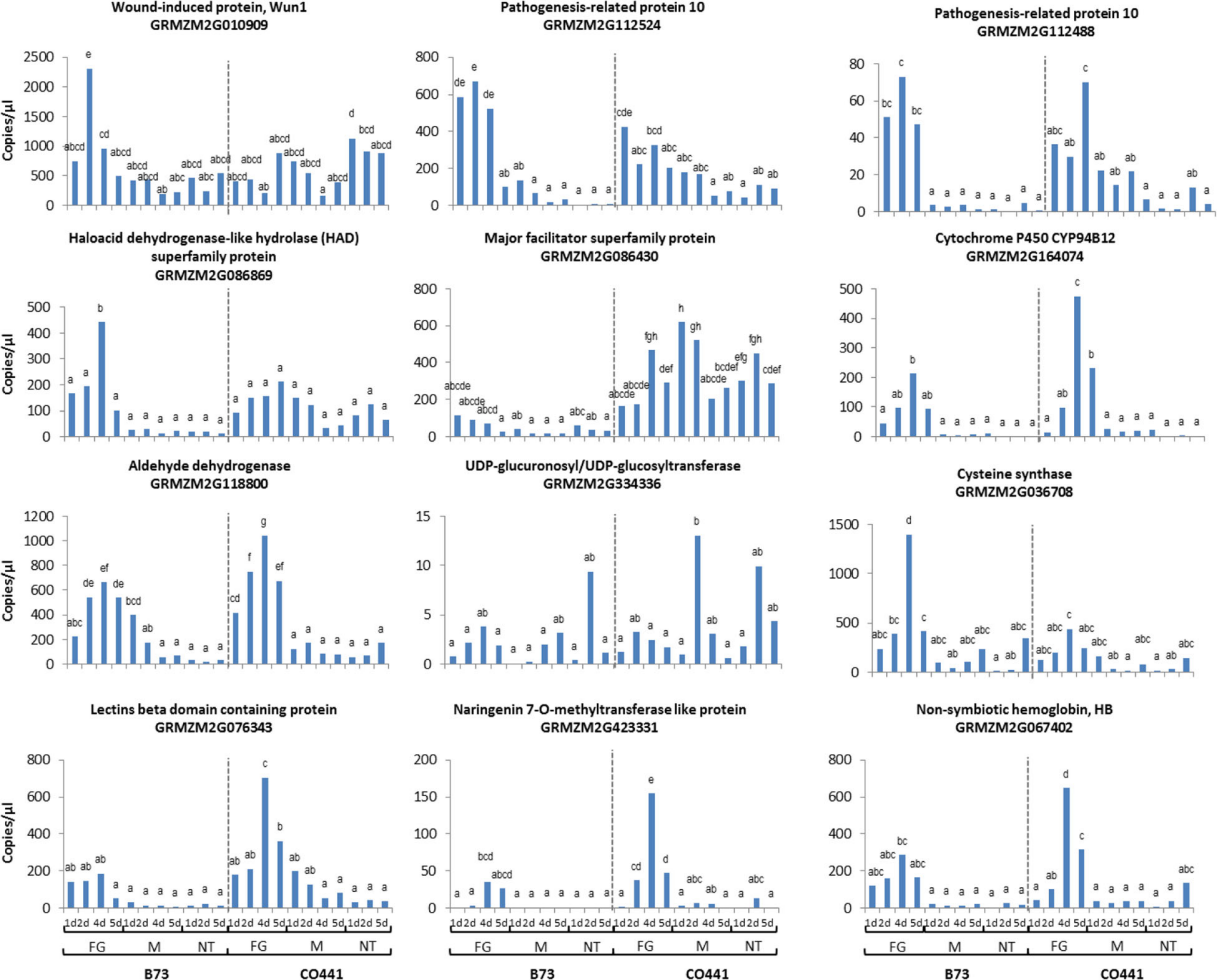
Validation of candidate gene expression profiles by droplet digital PCR. Candidate gene expression pattern 1, 2, 4 and 5 days after non-treated (NT), mock (M) and fungal (FG) inoculation of B73 and CO441 developing kernels using ddPCR. Each bar represent absolute expression averaged over three biological replicates. Letters above bars indicate least significant difference calculated from F-test

We further investigated the expression patterns of twelve selected genes using ddPCR over extended time points at 1, 2, 4 and 5 DAI (fungal and mock inoculated as well as untreated developing kernels) from field samples collected in 2013 (Fig. 5) in order to expand our gene expression profile information over a third field season. Five genes exhibited consistent expression profiles over the three field seasons. A MFS transporter (*GRMZM2G086430*) was induced at higher levels in the resistant inbred relative to B73 regardless of the treatment. Genes coding for a cytochrome P450 (CYP94B12; *GRMZM2G164074*), aldehyde dehydrogenase (GRMZM2G118800), methyltransferase (*GRMZM2G423331*), and a lectin beta domain containing protein (*GRMZM2G076343*) appeared to be more induced in CO441 with maximum expression levels attained at 4 DAI in CO441.

## Discussion

Our results showed that the sets of genes responding to fungal infection in a susceptible and a resistant inbred defined how each inbred perceived the intruder and programmed its defense system. After the host detected fungal attack, a series of signalling events took place which led to multiple oxidation-reduction processes and hormone biosynthesis that subsequently triggered or upregulated the expression of defense genes. Previously described defense genes encoding pathogenesis related proteins and phytoalexin biosynthetic enzymes [14, 35–37] were differentially expressed in response to fungal infection in both susceptible and resistant inbred lines. The ability to ultimately reduce disease severity appeared to be determined by the stronger, more constitutive expression of such defensive genes in the resistant inbred CO441 (Additional file 1: Table S4). Previous reports also showed that defensive genes expressed highly in CO441 were either fungal induced or expressed constitutively prior to pathogen attack [38, 39]. Maschietto and associates [40] observed that several genes coding for PR proteins or involved in oxidative stress responses exhibited higher expression levels in resistant (including CO441) relative to susceptible inbreds even prior to fungal inoculation.

Our RNA-Seq experiment complemented what had been previously documented by protein profiling of maize kernel tissue during *F. graminearum* infection [36]. When we compared the RNA-seq DEG with protein profiles [36] of the same two inbred lines, only ~ 4.3% of the DEG encoded proteins (52 of 1223) were detected in at least two biological replicates using iTRAQ protein profiling methods. Six DEG localized within GER resistance QTL regions and their encoded proteins were detected using both expression profiling methods (Additional file 1: Table S5).

We applied the criterion of differential expression to narrow the field of potential gene candidates lying within previously mapped resistance QTL regions. This method will overlook any gene whose expression is not significantly different between mock and fungal inoculation in either inbred. Using these parameters, we focused on 29 genes within QTL regions arising from the resistant inbred which were involved in signaling, cell wall modification, membrane transport, cell detoxification and biosynthesis of secondary metabolites.

We considered genes mapping within resistance QTL associated with both kernel inoculation and silk channel inoculation, as both inoculation methods ultimately lead to colonization of the developing ear. After silk channel inoculation, hyphae grow down silks to the ear, infecting kernels through the silk attachment point or by colonizing spaces between kernels and entering the rachis, spreading between kernels through the pedicel [41]. Kernel inoculation bypasses this journey between silk and kernel by mimicking infection facilitated by insect damage. However, mechanisms contributing to silk inoculation resistance could be found in the silk or other tissues in the path to colonize the ear including the developing kernels.

### Hormone signaling and modulation

The interplay of phytohormones influences the plant host response to fungal pathogens. The hormone jasmonic acid plays a role in early defence against *F. graminearum* in monocots including wheat and barley [12, 42]. One of the major QTL regions we identified on chromosome 2 harbors a cytochrome P450 gene (*GRMZM2G164074*; CYP94B12). Although this particular maize gene has not been characterized yet, members of the CYP94 family in Arabidopsis are involved in jasmonic acid turnover, mediating jasmonic acid inactivation and thereby controlling hormone levels [43, 44]. Pepper plants with a *cyp94b* gene disrupted were more susceptible to *Pseudomonas syringae*, a bacterial pathogen with a similar life style to *F. graminearum* [45]. As this maize CYP94B12 gene was found in a QTL region with the highest genetic effect for silk and second highest for kernel GER resistance [9] and both RNA-Seq and ddPCR data supported higher expression levels in CO441, it is possible that modulation of jasmonic acid may determine the efficacy of resistance. Chen and associates [12] reported ethylene as a susceptibility factor in wheat because they observed premature cell death in *F. graminearum* infected plants and found higher disease severity and conidia formation when ethylene was artificially applied in ethylene non-producing mutant wheat plants. *GRMZM2G086869* (haloacid dehalogenase-like hydrolase superfamily protein) within a QTL on chromosome 1 was also identified in a co-expression network tightly linked to ethylene-responsive genes during *A. flavus* infection [46].

### Secondary metabolites and defense proteins

After hormone biosynthesis, the downstream reaction of fungal infected host tissue would be either cell death of the infected tissue (hypersensitive response) and/or the production of secondary metabolites such as alkaloids, flavonoids, terpenoids (collectively known as phytoalexins) and pathogenesis related (PR) proteins. However, the hypersensitive response would not prevent *F. graminearum* from spreading but would instead support the necrotrophic stage of its life cycle. On the other hand, phytoalexins have been reported to directly exert antimicrobial activity on the fungus or create structural barriers by lignification of the plant cell wall [47].

One of the QTL regions we detected for GER resistance to silk channel inoculation on chromosome 9 harbored an uncharacterized gene, *GRMZM2G423331*, encoding a protein with 67% sequence similarity to narigenin 7-O-methyltransferase, an enzyme responsible for the biosynthesis of a rice phytoalexin called sakuranetin [48]. Sakuranetin is a well characterized flavonoid with negative effects on fungal spore germination [49]. Further characterization of this gene in maize could link *GRMZM2G423331* to the biosynthesis of known or novel phytoalexins or other secondary metabolites [13, 14, 37, 50, 51].

Three PR-10 genes, *GRMZM2G112538*, *GRMZM2G112524*, and *GRMZM2G112488*, were significantly up regulated within a major QTL region on chromosome 1. These genes encode small, acidic, intercellular proteins responsive to stress [52, 53]. The first two genes are almost identical to each other, sharing 98% nucleotide identity and coding for the same protein. The protein product of *GRMZM2G112538* and *GRMZM2G112524* was reported to inhibit fungal growth and conidia germination of *Aspergillus flavus*, another fungal pathogen causing maize ear rot [53, 54]. Significantly higher levels of these PR-10 proteins were observed in CO441 relative to B73 in our proteomics study [36].

Another candidate gene, *GRMZM2G010909*, was discovered in the same QTL region as the *PR-10* gene cluster. This gene encodes a protein with 64% amino acid similarity with a wound induced protein 1 in rice *Oryza sativa Japonica* and an orthologue was reported to provide structural reinforcement by accumulating in the cell wall of the ice plant [55]. Based on sequence similarity, we speculate that mechanical wounding or pathogen attack may have induced *GRMZM2G010909* in order to heal damaged tissue and/or, coupled with secondary metabolite fortification, modify the cell wall structure to create a barrier against fungal spread.

### Cell detoxification: Neutralization of DON and other xenobiotics

We found numerous candidate genes within QTL regions that are potentially involved in mycotoxin detoxification contributing to GER resistance. *F. graminearum* biosynthesizes a diverse array of secondary metabolites, including trichothecenes and butenolide. Trichothecenes such as deoxynivalenol (DON) damage host cell membranes, disrupt protein synthesis and accelerate host cell death [56]. We reported earlier that DON concentrations in maize kernels (2004 and 2006 field seasons) 2d post *F. graminearum* inoculation were about five-fold higher in B73 compared to CO441 [36]. Butenolide cytoxicity in animal cells is associated with glutathione depletion and lipid peroxidation [57, 58]. During the process of infection with other *Fusarium* species, oxidative degradation of lipid membranes was observed in susceptible maize but not the resistant CO441 inbred [39]. In barley, genes responding specifically to DON applications include glutathione S-transferases, cysteine synthase, and UDP-glucosyltransferase [59]. *GRMZM2G334336* (UDP-glucosyltransferase), *GRMZM2G036708* (cysteine synthase) and *GRMZM2G118800* (aldehyde dehydrogenase) were among the candidate genes we identified within a major QTL region on chromosome 2. One of the closest orthologues to *GRMZM2G334336* (encoded proteins share 70% amino acid identity) is the barley UDP-glucosyltransferase HvUGT13248 which was reported to convert DON to the less harmful compound DON-3-O-glucoside [60]. Cysteine synthase on the other hand may indirectly reduce the effect of DON by increasing cysteine and compensating for depleted glutathione [59]. Aldehyde dehydrogenase can metabolize toxic aldehydes and alleviate oxidative and/or osmotic stress [61]. Combinatory effects of enzymes, perhaps with promiscuous activities, could provide some protection against toxic fungal metabolites.

Phytoglobins influence programmed cell death (PCD) through nitric oxide scavenging and hormone modulation during embryogenesis and abiotic and biotic stress [62]. Improved resistance to the necrotroph *Verticillium dahliae* was observed following *Pgb1* overexpression in *Arabidopsis* [63]. Overexpression of *ZmPgb1.1* (*GRMZM2G067402*) in maize led to reduced reactive oxygen species production and increased tolerance to flooding stress [64]. We observe elevated levels of *ZmPgb1.1* in CO441 following *Fusarium* challenge (Fig. 5; Additional file 1: Table S5; Additional file 2), perhaps reducing oxidative damage in maize tissues and inhibiting the necrotrophic stage of *F. graminearum.*

*Tasselseed 2* (*GRMZM2G455809; TS2*) was previously found by SNP analysis to be significantly associated with resistance to Northern Leaf Blight caused by the hemibiotrophic fungal pathogen *Setosphaeria turcica* [65]. Encoding a short chain dehydrogenase with substrate specificity for certain steroid and dicarbonyl compounds, *TS2* is involved in sex determination cell death [66] and falls within a GER resistance QTL on chromosome 1.

The host cell detoxifies both internal and external xenobiotics and confines them internally in vacuoles or deposits them in apoplastic regions as bound residues, facilitated by membrane transporters [67]. Two classes of membrane-bound transporter proteins found in plants are the ATP- Binding Cassette (ABC) superfamily and Major Facilitator superfamily (MFS). A wheat ABC transporter (TaABCC3) was demonstrated to be associated with *F. graminearum* resistance [68]; however, there is no evidence that TaABCC3 is directly involved in DON transport and Walter and associates [68] speculate that TaABCC3 may be involved in chloroplast catabolite turnover, reducing DON-induced bleaching and stress. From our set of fungal responsive DEG, we identified an ABC transporter gene (*GRMZM5G803404;* ABCG subgroup) and two presumed MFS transporter genes (*AC208897.3_FGT004* and *GRMZM2G086430*) within GER resistance QTL regions on chromosomes 1 and 2. Notably, *GRMZM2G086430* is expressed at much higher levels in CO441 than B73 (Fig. 5, Additional file 1: Table S5).

## Conclusion

The current study characterized the plant response to Gibberella ear rot disease in maize using gene expression profiling of two inbred lines with contrasting levels of resistance and identified fungal responsive genes mapping within chromosomal regions associated with GER resistance. This information helped us to identify candidate genes that are possibly relevant in the defense response to help understand GER resistance mechanisms. Our study focused on genes represented in the B73 genome sequence and therefore any novel genes present in this germplasm were not considered as they could not be easily mapped to the genome. In particular, four genes on chromosome 2, which were consistently expressed at higher levels in the resistant inbred and lying within kernel resistance QTL regions, seemed the most promising, namely a MFS transporter, a cytochrome P450, an aldehyde dehydrogenase and a lectin domain gene. Further validation of the association of these genes with GER resistance QTLs is necessary to improve our understanding of maize resistance to *F. graminearum*.

## Additional files

Additional file 1: Table S1. Primers used for selected candidate and reference genes in droplet digital PCR validation experiment. **Table S2.** Hourly temperature readings during inoculation and tissue collection time periods in 2004, 2006, and 2013. **Table S3.** Mapping of RNA-Seq reads to the reference genome B73 V2. **Table S4.** List of significant differentially expressed transcripts. **Table S5.** Upregulated transcripts mapping within GER resistance QTL regions. (XLSX 1156 kb)

Additional file 2: Figure S1. Comparison between ddPCR and RNA-Seq expression profiles of selected genes. The Y-axis scale corresponds to transcripts per million (TPM) for RNA-Seq data and copies/μl for ddPCR. Tissue samples from the 2004 and 2006 field season were used for both gene expression quantitation methods. (PDF 288 kb)

## Abbreviations

DAI: Days after inoculation
ddPCR: Droplet digital polymerase chain reaction
DEG: Differentially expressed genes
FDR: False discovery rate
GER: Gibberella ear rot
QTL: Quantitative trait loci
SNP: Single nucleotide polymorphism
