# Supplementary material for: Transcriptome profiling of two maize inbreds with distinct responses to Gibberella ear rot disease to identify candidate resistance genes
Source: BMC Genomics. 2018 Feb 9;19:131. doi: 10.1186/s12864-018-4513-4 (PMC5807830; doi:10.1186/s12864-018-4513-4)
Supplement: Supplementary file 2 — Comparison between ddPCR and RNA-Seq expression profiles of selected genes. The Y-axis scale corresponds to transcripts per million (TPM) for RNA-Seq data and copies/μl for ddPCR. Tissue samples from the 2004 and 2006 field season were used for both gene expression quantitation methods. (PDF 288 kb) [file 12864_2018_4513_MOESM2_ESM.pdf]

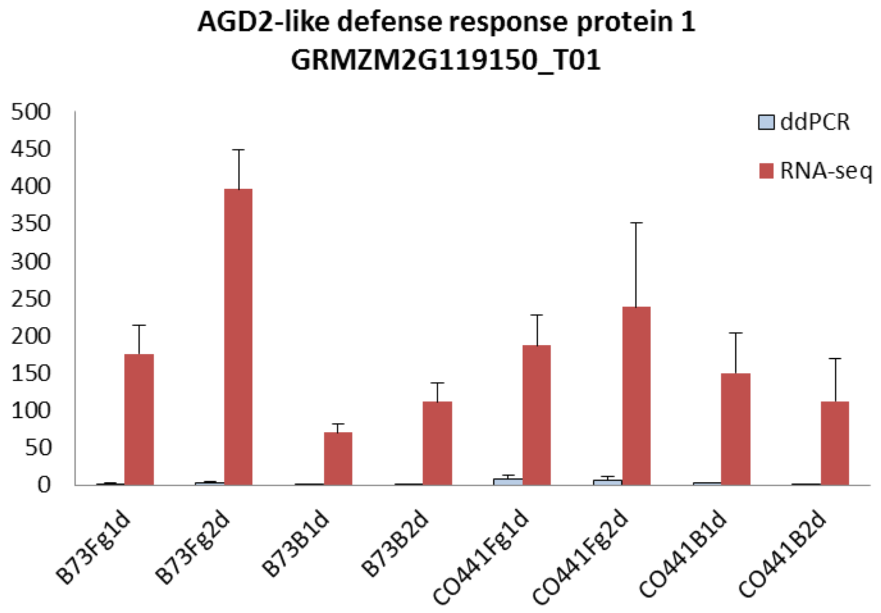
AGD2-like defense response protein 1
GRMZM2G119150_T01
500
450
400
350
300
250
200
150
100
50
0
ddPCR
RNA-seq
B73Fg1d
B73Fg2d
B73B1d
B73B2d
CO441Fg1d
CO441Fg2d
CO441B1d
CO441B2d

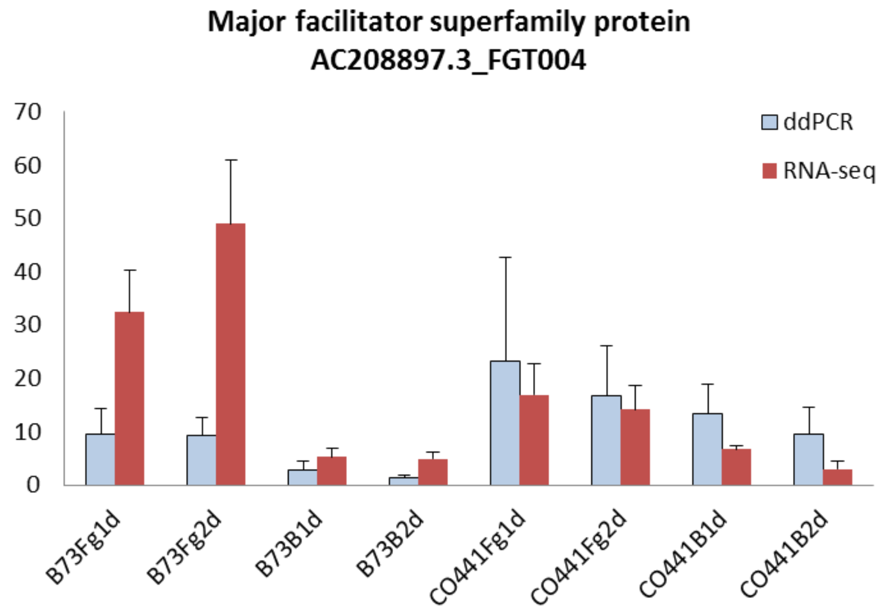
Major facilitator superfamily protein
AC208897.3_FGT004
70
60
50
40
30
20
10
0
ddPCR
RNA-seq
B73Fg1d
B73Fg2d
B73B1d
B73B2d
CO441Fg1d
CO441Fg2d
CO441B1d
CO441B2d

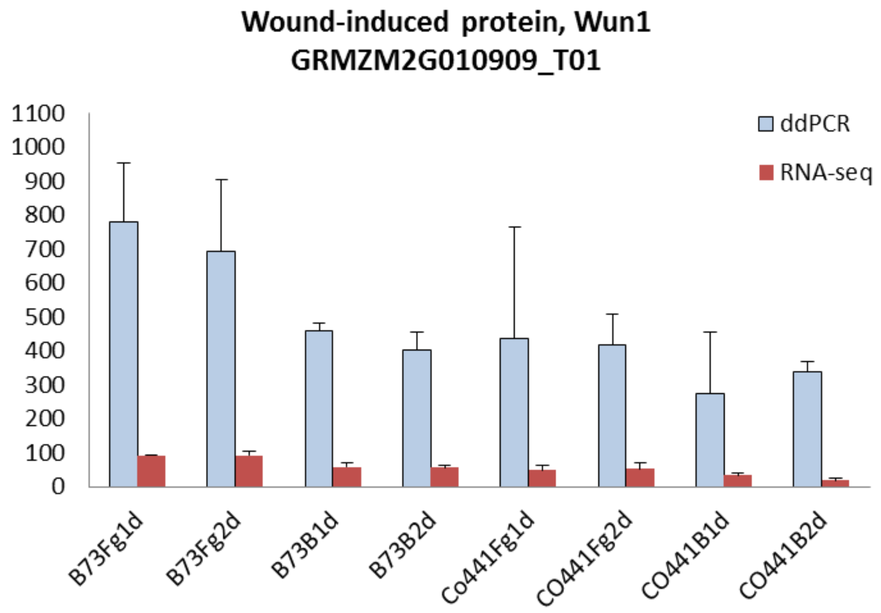
Wound-induced protein, Wun1
GRMZM2G010909_T01
1100
1000
900
800
700
600
500
400
300
200
100
0
ddPCR
RNA-seq
B73Fg1d
B73Fg2d
B73B1d
B73B2d
Co441Fg1d
CO441Fg2d
CO441B1d
CO441B2d

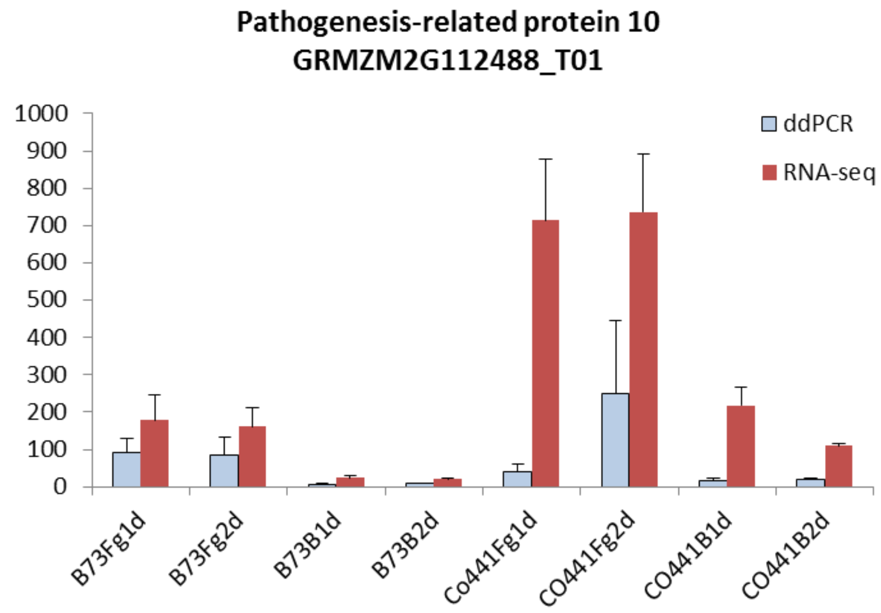
Pathogenesis-related protein 10
GRMZM2G112488_T01
1000
900
800
700
600
500
400
300
200
100
0
ddPCR
RNA-seq
B73Fg1d
B73Fg2d
B73B1d
B73B2d
Co441Fg1d
CO441Fg2d
CO441B1d
CO441B2d

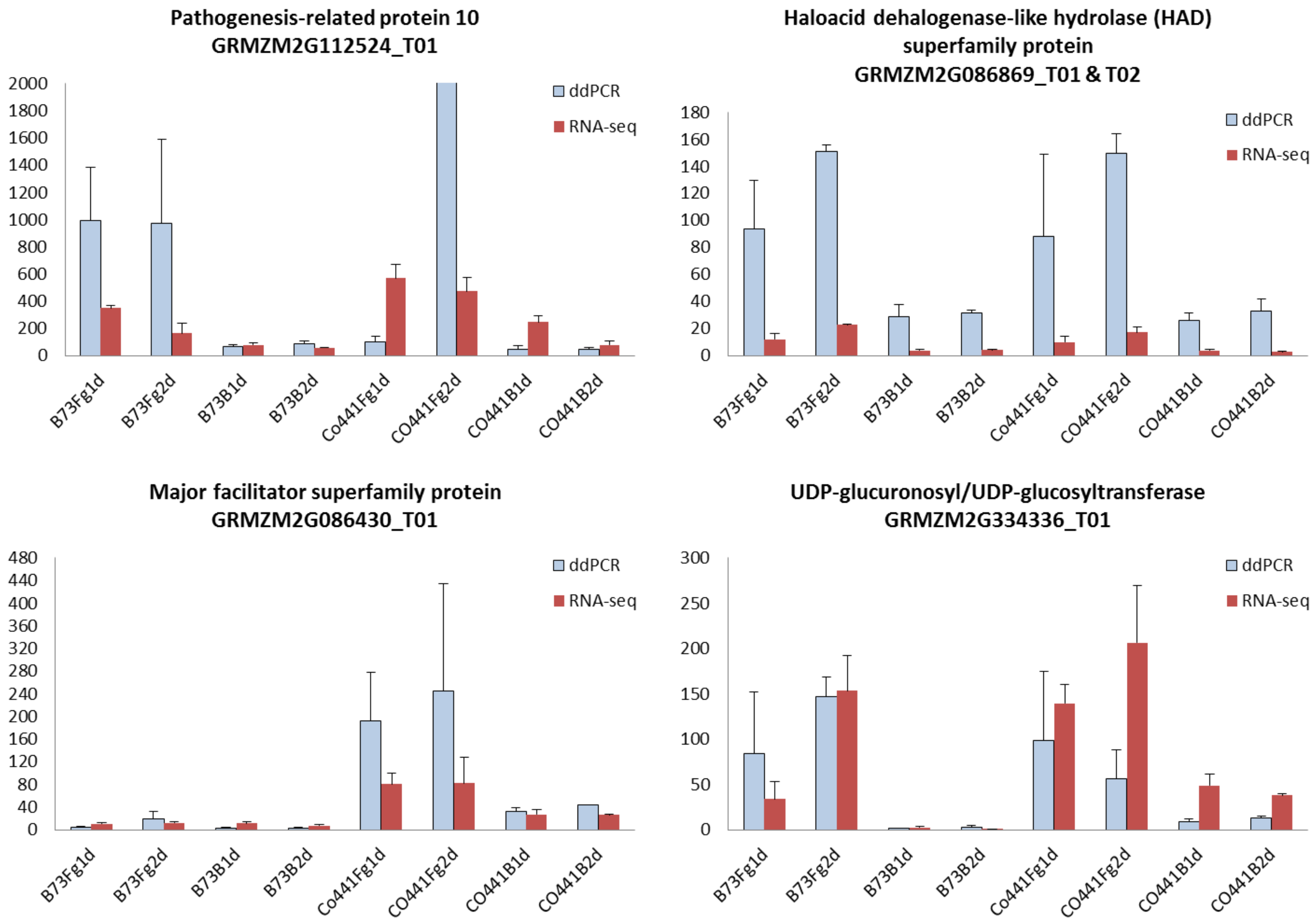
Pathogenesis-related protein 10
GRMZM2G112524_T01
ddPCR
RNA-seq
0
200
400
600
800
1000
1200
1400
1600
1800
2000
B73Fg1d
B73Fg2d
B73B1d
B73B2d
Co441Fg1d
CO441Fg2d
CO441B1d
CO441B2d
Haloacid dehalogenase-like hydrolase (HAD) superfamily protein
GRMZM2G086869_T01 & T02
ddPCR
RNA-seq
0
20
40
60
80
100
120
140
160
180
B73Fg1d
B73Fg2d
B73B1d
B73B2d
Co441Fg1d
CO441Fg2d
CO441B1d
CO441B2d
Major facilitator superfamily protein
GRMZM2G086430_T01
ddPCR
RNA-seq
0
40
80
120
160
200
240
280
320
360
400
440
480
B73Fg1d
B73Fg2d
B73B1d
B73B2d
Co441Fg1d
CO441Fg2d
CO441B1d
CO441B2d
UDP-glucuronosyl/UDP-glucosyltransferase
GRMZM2G334336_T01
ddPCR
RNA-seq
0
50
100
150
200
250
300
B73Fg1d
B73Fg2d
B73B1d
B73B2d
Co441Fg1d
CO441Fg2d
CO441B1d
CO441B2d

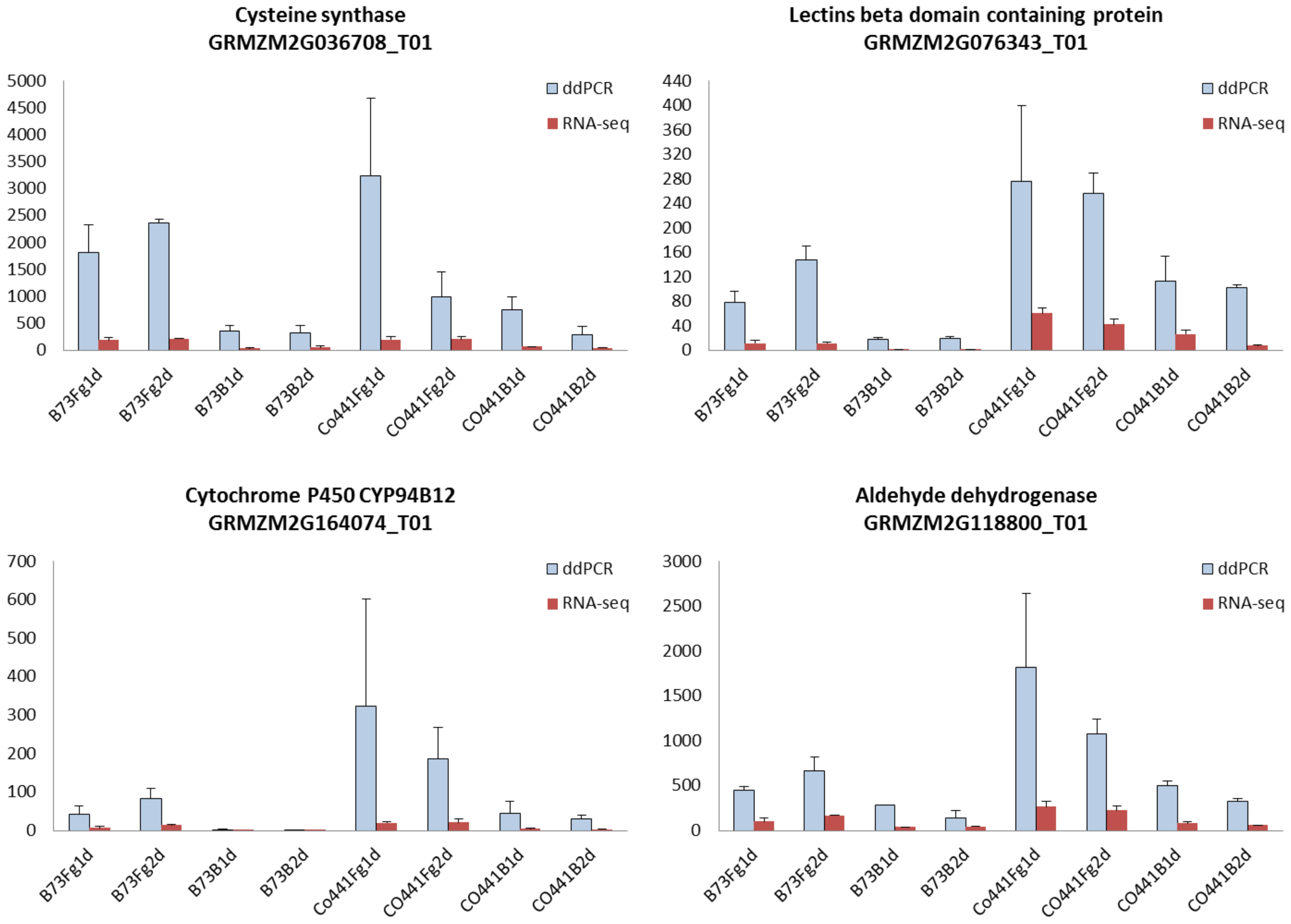
Cysteine synthase
GRMZM2G036708_T01
0
500
1000
1500
2000
2500
3000
3500
4000
4500
5000
ddPCR
RNA-seq
B73Fg1d
B73Fg2d
B73B1d
B73B2d
Co441Fg1d
CO441Fg2d
CO441B1d
CO441B2d
Lectins beta domain containing protein
GRMZM2G076343_T01
0
40
80
120
160
200
240
280
320
360
400
440
ddPCR
RNA-seq
B73Fg1d
B73Fg2d
B73B1d
B73B2d
Co441Fg1d
CO441Fg2d
CO441B1d
CO441B2d
Cytochrome P450 CYP94B12
GRMZM2G164074_T01
0
100
200
300
400
500
600
700
ddPCR
RNA-seq
B73Fg1d
B73Fg2d
B73B1d
B73B2d
Co441Fg1d
CO441Fg2d
CO441B1d
CO441B2d
Aldehyde dehydrogenase
GRMZM2G118800_T01
0
500
1000
1500
2000
2500
3000
ddPCR
RNA-seq
B73Fg1d
B73Fg2d
B73B1d
B73B2d
Co441Fg1d
CO441Fg2d
CO441B1d
CO441B2d

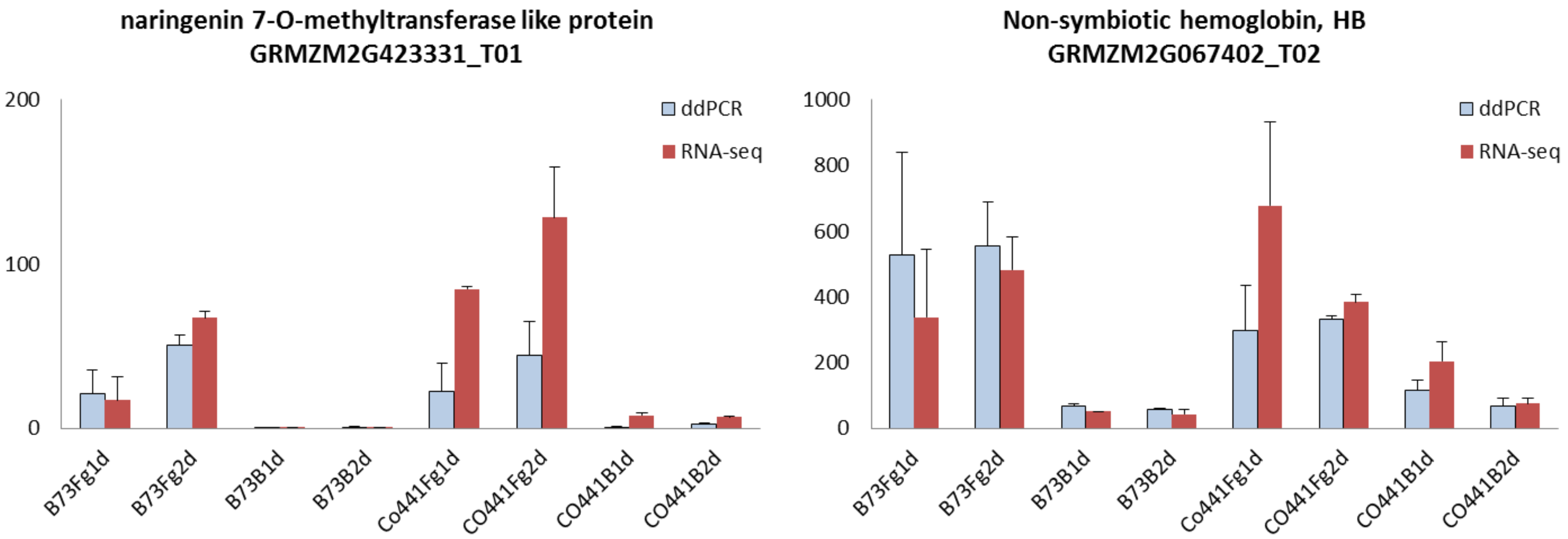

Supplementary Figure 1. Comparison between ddPCR and RNA-Seq expression profiles of selected genes.
The Y-axis scale corresponds to Transcripts Per Million (TPM) for RNA-Seq data and copies/µl for ddPCR. Tissue samples from the 2004 and 2006 field season were used for both gene expression quantitation methods. Each bar represents average of two biological replicates with standard error.
